# Safety and Efficacy of Neoadjuvant Therapy in Cholangiocarcinoma: Protocol for a Systematic Review and Meta-Analysis

**DOI:** 10.2196/84912

**Published:** 2026-06-10

**Authors:** Saikat Mandal, Arkadeep Dhali, Manideepa Maji, Suresh Vasan Venkatachalapathy, Amardeep Khanna, Dhanwant Gomez, Arvind Arora, Guruprasad Aithal

**Affiliations:** 1 Nottingham Digestive Diseases Centre, Translational Medical Sciences, School of Medicine, University of Nottingham, Nottingham, United Kingdom; 2 NIHR Nottingham Biomedical Research Centre, Nottingham University Hospitals NHS Trust and the University of Nottingham, Nottingham, United Kingdom; 3 W/E 1377, Nottingham Digestive Diseases Centre, E Floor, West Block, Queen’s Medical CentreNottingham, NG7 2UH, United Kingdom, (+44) 0115 970 9900 (Ext 80616); 4 Sheffield University Teaching Hospitals NHS Foundation Trust, Sheffield, United Kingdom; 5 Imperial College, London, United Kingdom; 6 Hull York Medical School, University of Hull, Hull, United Kingdom; 7 Hull University Teaching Hospitals NHS Trust, Hull, United Kingdom; 8 HPB medicine and Gastroenterology, Nottingham University Hospitals NHS Trust, Nottingham, United Kingdom; 9 HPB and GI Surgery, Nottingham University Hospitals NHS Trust, Nottingham, United Kingdom; 10 Medical Oncology, Nottingham University Hospitals NHS Trust, Nottingham, United Kingdom

**Keywords:** biliary tract cancer, cholangiocarcinoma, chemotherapy, neoadjuvant chemotherapy, bile duct cancer

## Abstract

**Background:**

Cholangiocarcinoma is an aggressive biliary tract malignancy with poor long-term survival and high recurrence rates even after apparently curative surgery. Although postoperative systemic therapy is incorporated into contemporary management, the role of neoadjuvant therapy remains uncertain because the available evidence is heterogeneous and largely observational and often mixes initially resectable and initially unresectable disease.

**Objective:**

This protocol describes a systematic review and meta-analysis that will evaluate the safety and efficacy of neoadjuvant therapy followed by curative-intent surgery or liver transplantation in adults with cholangiocarcinoma. The review will address 2 clinically distinct pathways: initially resectable disease, in which neoadjuvant therapy is intended to improve oncological outcomes, and initially unresectable disease, in which treatment is intended to downstage disease or bridge patients to potentially curative surgery or transplantation.

**Methods:**

The protocol has been prepared in accordance with the PRISMA-P (Preferred Reporting Items for Systematic Reviews and Meta-Analyses Protocols) 2015 statement and registered in PROSPERO. We will include randomized controlled trials and comparative nonrandomized studies evaluating neoadjuvant chemotherapy, chemoradiotherapy, immunotherapy, targeted therapy, or other preoperative antitumor therapy followed by curative-intent surgery or transplantation vs upfront surgery without preoperative systemic therapy. Single-arm studies, case reports, case series, and purely palliative cohorts will be excluded. The primary outcome will be overall survival analyzed as a time-to-event outcome using hazard ratios. Secondary outcomes will include recurrence-free survival, R0 resection, pathologic or radiologic complete response, conversion to operability among initially unresectable cohorts, and postoperative morbidity and mortality. Two reviewers will independently screen studies, extract data, and assess risk of bias using version 2 of the Cochrane risk-of-bias tool for randomized trials and the Risk of Bias in Nonrandomized Studies of Interventions tool for nonrandomized comparative studies. Random-effects meta-analysis; subgroup analyses; sensitivity analyses; assessment of reporting bias; and Grading of Recommendations Assessment, Development, and Evaluation (GRADE) certainty assessment will be undertaken where appropriate.

**Results:**

Database searches were completed on February 27, 2026. Title and abstract screening and full-text eligibility assessment were completed in March 2026. Data extraction commenced in April 2026. Meta-analysis is scheduled for May 2026, risk-of-bias and GRADE assessments are scheduled for June 2026, final manuscript preparation is scheduled for August 2026, and submission of the completed systematic review manuscript is scheduled for September 2026.

**Conclusions:**

This review will provide a methodologically explicit synthesis of comparative evidence on neoadjuvant therapy in cholangiocarcinoma while prespecifying separate analyses for initially resectable and initially unresectable disease. The findings are expected to inform future trial design, interpretation of emerging comparative evidence, and clinical decision-making in a setting where treatment intent varies substantially by resectability status.

## Introduction

### Background

Cholangiocarcinoma (CCA)—cancer of the bile duct (biliary tract)—affects approximately 3000 people in the United Kingdom each year; its incidence and mortality are increasing and now equal to that of hepatocellular carcinoma [1]. Often diagnosed at a late stage, CCA has a dismal prognosis, with only 13% surviving 3 years [1]. It is classified by anatomical site: perihilar and distal forms are present in 80% to 90% of patients (commonly known as extrahepatic CCA), and intrahepatic CCA occurs in 10% to 20% of patients [2,3]. The perihilar and distal subtypes present with narrowed segments (strictures) of the bile ducts, whereas intrahepatic CCA appears as focal lesions in the liver, each without specific diagnostic imaging features.

CCA is often diagnosed at a late stage due to nonspecific and nonbiliary obstructive symptoms. Conventional radiology cannot confirm diagnosis; it relies on histopathology, which has limited sensitivity due to the high heterogeneity of the tumor and spatial variability within areas of cancer [4,5]. It also becomes increasingly difficult to obtain samples for diagnostic microscopy in cases in which the bile duct is narrowed.

Surgical resection is the only potential curative treatment option for CCA. However, only 20% to 30% and 35% of patients present with surgically resectable disease in cases of intrahepatic CCA and perihilar CCA, respectively [6-8]. The recurrence rate of CCA remains high even following curative-intent surgical resection. Up to 25% of patients with CCA who undergo surgical resection develop very early recurrence within 6 months of surgery [9]. The European Society for Medical Oncology recommends capecitabine as adjuvant chemotherapy for early-stage CCA and cisplatin-gemcitabine for locally advanced cases [10]. The American Society of Clinical Oncology advises offering patients with resected biliary tract cancer adjuvant capecitabine chemotherapy for 6 months [11]. Unfortunately, none of these guidelines mentions or recommends neoadjuvant therapy. Recently, immune checkpoint inhibitors and a few targeted therapies have been approved as second-line therapies when CCA progresses under first-line treatment, including larotrectinib (NTRK fusion), pemigatinib (FGFR2 fusion or rearrangement), pembrolizumab (high microsatellite instability or mismatch repair deficiency), and ivosidenib (IDH1 R132 mutation) [10,11]. In our pilot search, it was noted that there are a lot of studies and some clinical trials (2 recently completed randomized controlled trials [RCTs]) that compare the efficacy of these immune checkpoint inhibitors, targeted therapies, and other therapies (eg, chemotherapies and radiochemotherapies) as neoadjuvant therapies prior to surgical intervention.

Given the poor prognosis, high risk of recurrence, and frequently delayed diagnosis associated with CCA, there is a critical need to develop effective adjuvant and neoadjuvant therapeutic strategies that can provide better complete responses (CRs) and delay the recurrence of malignancy. In this context, a systematic review and meta-analysis evaluating the safety and efficacy of neoadjuvant therapy in patients with CCA is both timely and feasible and could provide valuable guidance for clinical decision-making.

### Review Objectives

This systematic review aims to assess the safety and efficacy of neoadjuvant therapy (including preoperative chemotherapy, chemoradiotherapy, immunotherapy, and targeted therapy) in patients with CCA.

## Methods

This protocol was prepared in accordance with the PRISMA-P (Preferred Reporting Items for Systematic Reviews and Meta-Analyses Protocols) 2015 statement [12] and registered in PROSPERO (CRD420251111270). Any important protocol amendments will be documented in the final review manuscript.

### Types of Studies

We will include RCTs and comparative nonrandomized studies, including prospective and retrospective cohort studies (Textbox 1). Single-arm clinical trials, case reports, case series, narrative reviews, editorials, and studies limited to palliative treatment without a curative-intent pathway will be excluded. Conference abstracts and registry records will be used to identify potentially relevant studies, but only comparative studies with sufficient outcome data will be included in the synthesis.

Textbox 1. **Criteria for considering studies for this review.**

**Inclusion criteria**

- Types of studies: randomized controlled trials and comparative nonrandomized studies, including prospective and retrospective cohort studies
- Types of participants: adults (18 y or older) with intrahepatic, perihilar, or distal cholangiocarcinoma; initially resectable disease eligible for upfront curative-intent surgery or transplantation; and initially unresectable or borderline resectable disease only when neoadjuvant therapy is used with an explicit curative-intent downstaging or bridging pathway
- Intervention: preoperative chemotherapy, chemoradiotherapy, immunotherapy, targeted therapy, or other antitumor therapy before curative-intent surgery or liver transplantation
- Comparator: upfront surgery or liver transplantation without preoperative systemic therapy

**Exclusion criteria**

- Single-arm clinical trials and other noncomparative studies
- Case reports, case series, narrative reviews, editorials, and purely palliative cohorts
- Studies without a curative-intent pathway
- Conference abstracts or registry records without sufficient comparative outcome data for synthesis

### Types of Participants

Studies will be eligible if they include adults (aged 18 years or older) with histologically, cytologically, or otherwise clinically confirmed CCA of any anatomical subtype, including intrahepatic, perihilar, and distal CCA.

We will include studies that involve any of the following two clinically distinct participant groups: (1) patients with initially resectable disease who are candidates for upfront curative-intent surgery or transplantation and (2) patients with initially unresectable or borderline resectable disease in whom neoadjuvant therapy is used with the explicit intent of downstaging disease or bridging patients to curative-intent resection or transplantation (initially unresectable cohorts will be included only when the study clearly describes a curative-intent pathway after neoadjuvant therapy rather than purely palliative treatment).

### Types of Interventions

Eligible interventions will include preoperative chemotherapy, chemoradiotherapy, immunotherapy, targeted therapy, or other antitumor therapy administered before curative-intent surgery or liver transplantation. The comparator will be upfront surgery or liver transplantation without preoperative systemic therapy.

### Outcomes

#### Primary Outcomes

The primary outcomes will include the following:

- Overall survival: the time from randomization to death or last follow-up for alive patients.
- CR: CR was defined as the complete disappearance of all target lesions without any residual lesions. It may be a pathological CR confirmed histologically or a radiological CR assessed via imaging modalities such as positron emission tomography, magnetic resonance imaging, or computed tomography.

#### Secondary Outcomes

The secondary outcomes will include the following:

- Patients with postoperative outcomes of surgical R0 resection
- Relapse-free survival: time from surgery to disease recurrence or death in patients who undergo surgery with curative intent
- Patients with postoperative complications

We will not use the outcomes above as criteria for including or excluding studies (ie, studies will not be excluded for not reporting a particular outcome). However, these outcomes will serve as the basis for data extraction and analysis.

### Search Methods for the Identification of Studies

#### Electronic Searches

We will conduct a comprehensive search of major biomedical databases for eligible studies with no restrictions on language or publication date. The following databases will be searched from their inception to the present:

- CENTRAL: for clinical trial reports and relevant systematic review references (latest issue)
- MEDLINE (via Ovid or PubMed; 1946 to the present): to capture published literature in medicine
- Embase (Ovid; 1974 to the present): to ensure coverage of European and additional biomedical literature not indexed in MEDLINE
- CINAHL (1937 to the present): for any nursing or allied health journals that might include CCA-related studies
- LILACS (1982 to the present): for literature from Latin America
- Web of Science Conference Proceedings Citation Index (Science; 1990 to the present): to capture abstracts from major scientific conferences in case relevant trial results in CCA have been reported in conference proceedings
- Scopus (1960 to the present): to provide comprehensive coverage of peer-reviewed journals across multidisciplinary biomedical and life sciences literature, capturing relevant publications on neoadjuvant chemotherapy, radiotherapy, immunotherapy, and hepato-pancreato-biliary malignancies not exclusively indexed in MEDLINE or Embase
- ClinicalTrials.gov and the World Health Organization International Clinical Trials Registry Platform: for ongoing or recently completed trials that may not yet be published

Our search strategy will use combinations of terms for CCA (eg, “Bile Duct Neoplasms” and “Bile Duct Cancer”) and neoadjuvant therapy (eg, “Neoadjuvant Therapy,” “Neoadjuvant chemotherapy,” “Neoadjuvant immunotherapy,” “Neoadjuvant radiotherapy,” “Cis/Gem,” “Neoadjuvant Capecitabine,” “Gem Cis durvalumab,” “GEMOX,” “Neoadjuvant CapOx,” and “Neoadjuvant Gemcitabine”) in various spellings. Because we aim to include both RCTs and observational studies, we will not apply a study design filter that could exclude nonrandomized studies. In databases that filter (eg, MEDLINE), we will avoid the highly sensitive RCT filter to retain observational evidence. Search strategies will be developed in consultation with an information specialist and peer reviewed in accordance with best practices (eg, PRESS [Peer Review of Electronic Search Strategies] guidelines). A full MEDLINE and Embase search strategy illustrating the combination of MeSH (Medical Subject Headings) terms and keywords has been provided in Multimedia Appendix 1. We will also ensure that our search covers any relevant non–English-language literature (any language), and if needed, we will have non–English-language studies translated or assessed by persons proficient in that language.

#### Searching Other Resources

In addition to electronic databases, we will search other resources to identify unpublished or ongoing studies. We will hand search the reference lists of all included studies and relevant review articles for additional citations not captured by database searches. We will contact content experts and the corresponding authors of included trials or studies to inquire about any unpublished data or ongoing studies. We will contact them at least twice before declaring them as nonresponders with an interval of 10 working days. Where applicable, we will review conference abstract books (eg, American Society of Clinical Oncology or European Society for Medical Oncology) and proceedings for the past 5 years to identify any trials in progress or results reported in abstract form. If we identify a conference abstract, trial registry entry, or other report of a study that lacks a full publication, we will attempt to contact the study investigators for further information or data.

We will also search regulatory agency websites and thesis or dissertation databases (eg, ProQuest) to ensure that we capture any relevant trials or observational studies in gray literature. All references identified through these sources will be imported into a reference management software, and duplicates will be removed prior to screening.

### Data Collection and Analysis

#### Selection of Studies

We will follow a 2-stage screening process to select studies as recommended by the *Cochrane Handbook for Systematic Reviews of Interventions*. First, 2 reviewers will independently screen all titles and abstracts yielded by the search against the predefined eligibility criteria. We will obtain the full text of any article that appears to meet the inclusion criteria or for which eligibility is uncertain. The 2 reviewers will then independently assess the full-text articles for inclusion using a standard eligibility checklist. Any disagreements or uncertainties regarding a study’s eligibility will be resolved through discussion. If consensus cannot be reached, a third reviewer will act as an arbiter. We will record the reasons for excluding any studies at the full-text screening stage.

**Figure 1.**
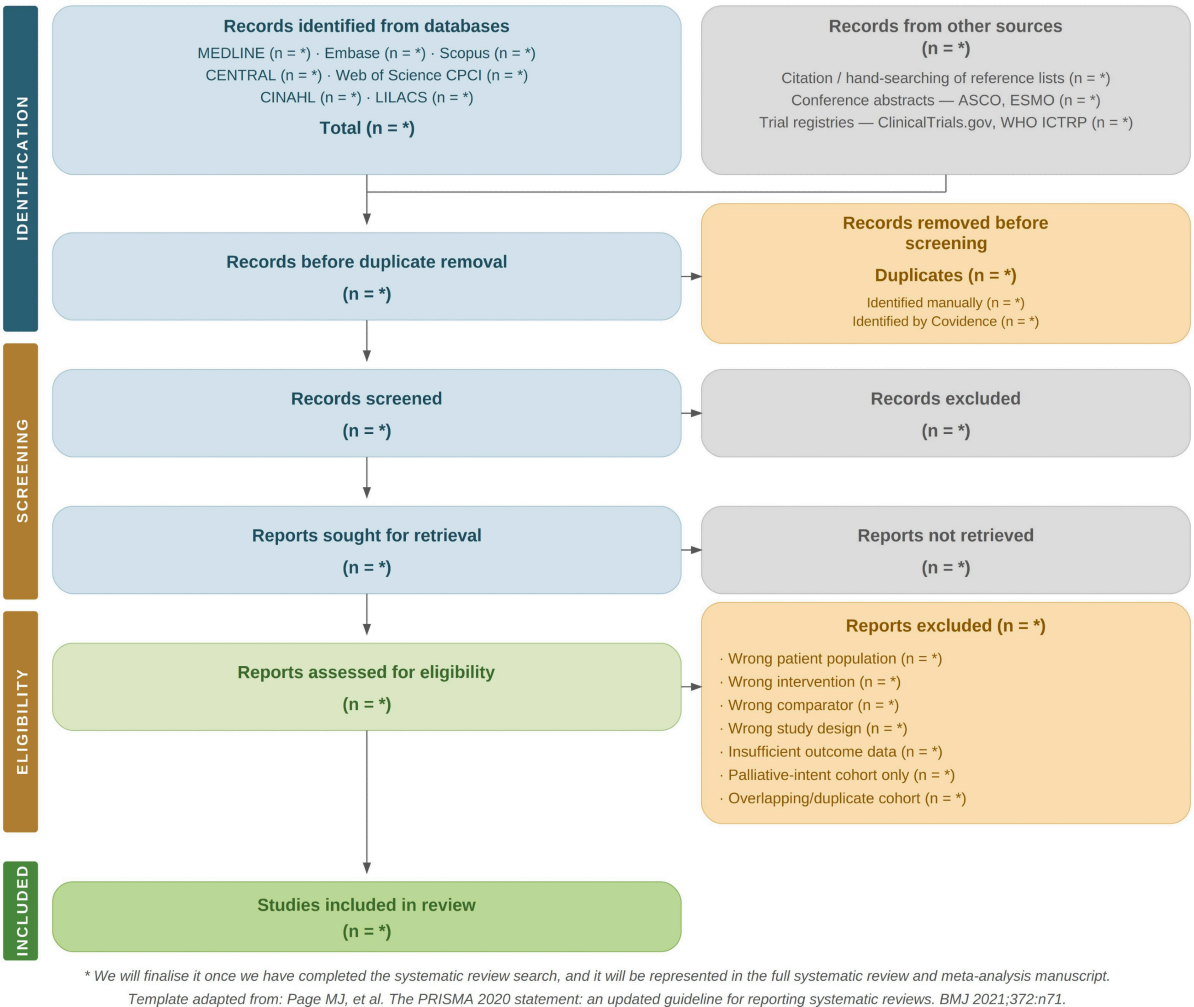
Prototype PRISMA (Preferred Reporting Items for Systematic Reviews and Meta-Analyses) 2020 flow diagram for the planned study selection process.

The completed systematic review will document the study selection process in a PRISMA (Preferred Reporting Items for Systematic Reviews and Meta-Analyses) 2020 flow diagram (Figure 1) detailing the number of records identified, screened, excluded, and included. All excluded studies at the full-text screening stage will be listed in a table with the reasons for exclusion.

#### Data Extraction and Management

Using a standardized data extraction form (developed a priori), 2 review authors will independently extract data from each included study. Before starting data extraction for all studies, we will pilot the data extraction form on a sample of studies (eg, 2 included trials) to ensure consistency and completeness, refining the form as necessary. Each reviewer will extract data, and a cross-check will be performed to compare entries. Disagreements or discrepancies in data extraction will be resolved through discussion between the 2 reviewers; if any issues remain unresolved, a third reviewer will be consulted to arbitrate. We will not be blinded to study authors or journals during data extraction as blinding is not practicable, and all included studies will undergo a full risk-of-bias assessment.

For each included study, we will extract the following information:

- Study identification and design: first author, year of publication, and journal or source; study design (eg, RCT or prospective cohort study); single center or multicenter; country and setting; duration of study and follow-up period; and any noted funding sources or conflicts of interest
- Participants: inclusion and exclusion criteria of the study, number of participants randomized or enrolled (and number analyzed), baseline demographic data (age and sex distribution), primary tumor origin of CCA (intrahepatic or extrahepatic), Charlson-Deyo comorbidity score, tumor level (ie, American Joint Committee on Cancer clinical T and N stages), and lympho-vascular invasion (absent or present)
- Interventions: details of neoadjuvant treatment (chemotherapy, targeted therapy, or immunotherapy), details of surgery, tumor resectability, and risk factors for recurrence
- Outcomes: all relevant outcome measures as listed above (eg, overall survival and relapse-free survival), as well as the numerical results for each outcome (eg, means and SDs for continuous outcomes; event counts and denominators for dichotomous outcomes; or hazard ratios [HRs] for time-to-event outcomes, such as survival, if reported), the time point at which the outcome was measured, and any definitions or criteria used (eg, how “relapse-free survival” was defined), first calculating the overall pooled analysis for CCA and then the subgroup analysis stratified by intrahepatic and extrahepatic CCA
- Risk of bias–related information: details relevant to assessing study quality, such as methods of random sequence generation and allocation concealment (for RCTs); blinding of participants, personnel, and outcome assessors; completeness of outcome data (attrition rates); and selective reporting of outcomes, as well as information on how confounding was addressed and whether any matching or adjustment for baseline differences was performed for observational studies
- Other: any notable findings or observations not captured by the data types above (eg, authors’ noted limitations or post hoc analyses) and any reported subgroup analyses within the study, as well as whether there was any early stopping (in RCTs) or any deviations from the protocol that could affect outcomes

All extracted data will be managed using Review Manager (The Cochrane Collaboration) or other appropriate software. One reviewer will enter the data into Review Manager, and a second reviewer will cross-check the entries for accuracy. If information is missing or unclear, we will contact the study authors for clarification. In the event of multiple publications or reports from the same study, we will extract data from all available sources to ensure completeness and use the most comprehensive or final dataset for analysis (while noting any discrepancies among sources).

#### Assessment of Risk of Bias in the Included Studies

Two review authors will independently assess the risk of bias for each included study using prespecified tools according to study design. For RCTs, we will use version 2 of the Cochrane risk-of-bias tool for randomized trials [13], which assesses bias in the randomization process, deviations from intended interventions, missing outcome data, measurement of outcomes, and selection of the reported results. Each domain, and the overall study result, will be rated as “low risk of bias,” “some concerns,” or “high risk of bias” in accordance with guidance from this tool. For nonrandomized comparative studies, including retrospective and prospective observational cohort studies, we will use the Risk of Bias in Nonrandomized Studies of Interventions tool [14]. This tool evaluates bias due to confounding, participant selection, classification of interventions, deviations from intended interventions, missing data, measurement of outcomes, and selection of the reported results. Each domain will be rated as having a low, moderate, serious, or critical risk of bias, and an overall risk-of-bias judgment will be assigned for each study. Any disagreements between reviewers will be resolved through discussion, with consultation with a third reviewer if necessary. The risk-of-bias assessments will be incorporated into the interpretation of findings and will inform the Grading of Recommendations Assessment, Development, and Evaluation (GRADE) assessment of certainty of evidence [15].

#### Measures of Treatment Effect

For each outcome, we will extract or calculate summary measures of effect comparing neoadjuvant therapy followed by surgery with upfront surgery, and we will analyze resectable and initially unresectable CCA separately. For dichotomous outcomes, such as R0 resection and postoperative complications, we will calculate risk ratios with 95% CIs. If events are very rare, we may use the Peto odds ratio method as appropriate for sparse data. Time-to-event outcomes, including overall survival and relapse-free or disease-free survival, will be analyzed primarily using HRs with 95% CIs. If HRs are not directly reported, we will derive or approximate them from other available summary statistics or survival curves using established methods [16,17]. For continuous outcomes, where applicable, we will use mean differences (MDs) with 95% CIs when studies report outcomes on the same scale or standardized MDs with 95% CIs when different instruments are used to measure the same construct.

If continuous data are reported as medians with IQRs, ranges, or both, we will estimate corresponding means and SDs using established statistical methods where appropriate [18,19]. If conversion is not feasible or is judged methodologically inappropriate, these results will be summarized narratively. When outcome data cannot be meaningfully pooled because of incompatible reporting formats, insufficient data, or substantial clinical or methodological heterogeneity, we will provide a qualitative synthesis instead of a pooled effect estimate.

#### Unit-of-Analysis Issues

We anticipate that the unit of analysis will be the individual patient in all included studies. Cluster randomized trials or crossover trial designs are unlikely in this context (it is improbable to cluster by center or to cross patients over between direct surgery and neoadjuvant therapy). If we do encounter a cluster randomized trial, we will ensure appropriate analysis by checking that clustering has been accounted for in the published analysis or by adjusting the effective sample size using intracluster correlation coefficients if necessary according to the Cochrane handbook. For any crossover trials (also unexpected in this context), we will use data from the first phase only (to avoid carryover effects) or otherwise treat them cautiously as outlined in the handbook. If multiple observations per participant are reported (eg, repeated measurements over time), we will either choose a consistent time point for analysis or use methods for longitudinal data if a meta-analysis is feasible, ensuring that each participant’s data are analyzed only once for each outcome. In summary, any unit-of-analysis issues identified will be handled in accordance with Cochrane recommendations to avoid unit-of-analysis errors.

#### Dealing With Missing Data

We will contact the study authors to request any missing outcome data or information that could not be obtained from the published report. If important data remain missing despite our requests, we will assess the potential impact on the findings. Where applicable, we will perform analyses on an intention-to-treat basis. For dichotomous outcomes, we will assume that participants lost to follow-up did not experience the event unless this is clearly inappropriate. For continuous outcomes, if only a per-protocol or completer analysis is available, we will note that and use whatever data are reported. We will record the extent of incomplete outcome data for each study (eg, numbers lost to follow-up or withdrawn) and consider how the study authors dealt with missing data. If feasible, we may conduct sensitivity analyses to assess the robustness of the results to assumptions about missing data (eg, by comparing best- and worst-case scenarios for dichotomous outcomes). We will not impute missing outcome data ourselves except for standard methods of calculating missing SDs (eg, from SEs or CIs) when necessary for meta-analysis following guidance in the Cochrane handbook.

#### Assessment of Heterogeneity

We will assess heterogeneity among studies by examining the clinical and methodological characteristics of the included trials and through statistical measures. If studies are sufficiently similar in terms of participants, interventions, outcomes, and timing, we will consider pooling their results in a meta-analysis. Statistical heterogeneity will be evaluated using the chi-square test (with a significance threshold of *P*<.10 indicating potential heterogeneity) and the *I*^2^ statistic. The *I*^2^ value describes the percentage of total variation across studies that is due to heterogeneity rather than chance. As a rough guide, we will consider an *I*^2^ value of more than 50% to represent moderate heterogeneity and of more than 80% to represent substantial (or considerable) heterogeneity. If heterogeneity is detected (chi-square *P*<.10 and/or *I*^2^>50%), we will first examine possible sources by considering differences in study design, populations, interventions, or risk of bias.

Given the likely variability in patient populations (stages and site of the malignancy) and neoadjuvant treatment protocols across studies, we anticipate at least moderate heterogeneity a priori. Therefore, we plan to use a random-effects model for meta-analysis by default, which is more appropriate when treatment effects may vary between studies. A random-effects model (eg, the DerSimonian-Laird method) [20] provides an average treatment effect while accounting for between-study variability. If heterogeneity is extremely high (*I*^2^>80%) and cannot be readily explained, we may refrain from computing an overall pooled effect and instead provide a descriptive summary of results. In such cases, we will still attempt to explore heterogeneity via subgroup analyses (see below) to determine whether specific subgroups of studies are more homogeneous.

#### Assessment of Reporting Biases

If we include 10 or more studies in a meta-analysis for a given outcome, we will assess potential publication bias (small-study effects) using a funnel plot. We will visually inspect funnel plots for asymmetry. Additionally, we will perform statistical tests for funnel plot asymmetry (such as the Egger regression test) [21] when appropriate, with a significance level of a *P* value below .10 as recommended for these tests due to their low power. If asymmetry is detected, we will consider possible reasons (eg, true heterogeneity, selective nonreporting of small studies, or publication bias).

Statistical tests and graphical methods used to detect reporting bias (such as funnel plots and the Egger test) have very low power and reliability when fewer than 10 studies are available. In this situation, true biases may easily go undetected, but potential bias remains a real concern as each study represents a large portion of the total. To resolve this, we will use qualitative judgment, such as providing a narrative assessment based on the context, study designs, and comprehensiveness of the search. We will also use structured tools such as the Risk of Bias Due to Missing Evidence tool [22] and document limitations.

#### Data Synthesis

We will conduct meta-analyses using the Review Manager software [23]. A meta-analysis will be carried out if multiple studies are sufficiently homogeneous (clinically and methodologically) to justify a combined quantitative synthesis. For dichotomous outcomes, we will typically use the Cochran-Mantel-Haenszel method [24] to calculate pooled risk ratios or odds ratios with 95% CIs. For continuous outcomes, we will use the inverse variance method to pool MDs or standardized MDs. Time-to-event outcomes will be combined using the generic inverse variance method (incorporating log HRs and their SEs). As noted, we will use a random-effects model [20] for primary analyses to account for between-study variation; however, we will also check results under a fixed-effects model in a sensitivity analysis to see whether conclusions differ. All analyses will be 2 tailed with a threshold of a *P* value below .05 for statistical significance unless otherwise specified.

If meta-analysis is not possible (eg, due to an insufficient number of studies or excessive heterogeneity), we will provide a narrative synthesis of the results. This will involve describing and tabulating study findings and possibly grouping studies by conceptual categories (such as site and stage of CCA or neoadjuvant treatment used) to qualitatively summarize patterns. Where outcomes are reported without adequate detail for analysis (eg, “improved” vs “not improved” without numeric data), we will attempt to obtain additional data from the authors or, if unavailable, report the outcomes narratively.

We will prepare a summary of findings table for the main comparison (neoadjuvant therapy followed by surgery to upfront surgery for treating patients with CCA) and key outcomes following the GRADE approach to assess the certainty of evidence. Outcomes in the summary of findings table will likely include patients with R0 resection, occurrence of complications, relapse-free survival, and overall survival, among others, as mentioned in the Outcomes section (as these reflect both benefits and harms and are of high importance to decision-makers). We will use the GRADEpro software (McMaster University and Evidence Prime) to construct this table and will justify any downgrading or upgrading of evidence quality in footnotes according to the GRADE [15] criteria (risk of bias, inconsistency, indirectness, imprecision, and publication bias).

#### Subgroup Analysis and Investigation of Heterogeneity

If sufficient data are available, we will perform subgroup analyses to explore potential sources of heterogeneity and examine effects in specific subpopulations. Anticipated subgroup analyses include the following.

##### Anatomical Site of the Malignancy: Intrahepatic or Extrahepatic CCA

This analysis will compare intrahepatic vs extrahepatic CCA based on the anatomical site of the malignancy. If we find further heterogeneity in the extrahepatic CCA group, we will analyze it further in the distal and hilar subgroups. Surgical and treatment approaches differ depending on anatomical site, which may result in different clinical outcomes.

##### Neoadjuvant Treatment Regimen

. Neoadjuvant targeted therapies and immunotherapy, as documented in different studies, include toripalimab and lenvatinib with chemotherapy or pembrolizumab or durvalumab with or without chemotherapy. Less frequently, studies will evaluate neoadjuvant radiotherapy. These chemotherapy, immunotherapy, and radiotherapy regimens have different adverse effects and vary widely in reported efficacy. Thus, we will conduct a separate outcome analysis for chemotherapy vs chemotherapy plus targeted therapy vs radiotherapy-based approaches.

##### Stages of CCA

Treatment outcomes and quality of life are expected to vary by stage of malignancy. For early-stage localized cancer, there will be a good number of patients who will achieve R0 resection; as a result, the overall survival will be different. Thus, we will perform a subgroup analysis of early-stage vs locally advanced CCA cases as reported in the studies.

##### Study Design

This comparison involves RCTs vs nonrandomized studies. We will compare results from high-quality RCTs to those from observational studies to see whether there are consistent findings. We will be cautious in interpreting subgroup differences by design as observational studies may be included primarily when RCT evidence is lacking.

##### Type of Surgery

This comparison will focus on transplantation vs resection. Liver transplantation has its own complications related to anastomosis, blood loss, rejection, and immunosuppression. Thus, the complication and effectiveness profiles of studies comparing transplantation to surgery will differ from those of simple resection.

##### Approach for Subgroup Analysis

For each subgroup analysis, we will perform stratified meta-analyses and, if appropriate, formally test for subgroup differences by conducting a chi-square test for subgroup interaction (as implemented in Review Manager). However, we acknowledge that these subgroup analyses are exploratory and may be underpowered. We will interpret them with caution, emphasizing where they generate hypotheses for further research.

### Sensitivity Analysis

We will conduct sensitivity analyses to test the robustness of our review findings to key methodological decisions and assumptions.

#### Exclusion of Studies With High Risk of Bias

We will repeat the primary analyses including only studies judged to have an overall low risk of bias (for RCTs, those with low risk in domains such as randomization and allocation concealment; for observational studies, those that adequately addressed confounding and other bias domains). This will show whether any particular risk of bias drives the results.

#### Exclusion of Studies With High Attrition

We will perform analyses excluding studies with high attrition (eg, more than 20% of participants not accounted for in the primary outcome analysis). This will test whether the results are sensitive to the inclusion of studies with potential attrition bias.

#### Analysis of RCTs Only

If our review includes both RCTs and observational studies, we will compare the results of an analysis including all eligible studies to those of an analysis restricted to RCTs. This will help determine whether the inclusion of nonrandomized data influences the effect estimates.

#### Fixed-Effects vs Random-Effects Model

We will compare the meta-analysis results using a fixed-effects model to our default random-effects approach to assess the impact of model choice on the conclusions, especially in the presence of few studies or when heterogeneity is low.

#### Approach for Sensitivity Analysis

For all sensitivity analyses, we will note any changes in the magnitude or significance of the effect estimates. If the conclusions remain consistent across these analyses, this will increase confidence in the robustness of the findings. Conversely, if results differ substantially under different assumptions, we will discuss these differences and their implications for the certainty of the evidence in our review.

### Ethical Considerations

Systematic reviews and meta-analyses are exempt from ethics approval and do not require participant consent as they use secondary data.

## Results

We received UK Research and Innovation funding in February 2025. The protocol was prospectively registered in PROSPERO, and database searches were completed on February 27, 2026. Title and abstract screening and full-text eligibility assessment were completed in March 2026. Data extraction commenced in April 2026. Meta-analysis is scheduled for May 2026, risk-of-bias and GRADE assessments are scheduled for June 2026, final manuscript preparation is scheduled for August 2026, and submission of the completed systematic review manuscript is scheduled for September 2026. As this is a protocol for a review of published and registered studies, no participants will be directly recruited.

A prototype PRISMA 2020 flow diagram is presented in Figure 1 to show the prespecified study selection pathway and the planned categories for exclusion. Numerical counts will be inserted after final verification of the study selection dataset and will be fully reported in the completed systematic review and meta-analysis manuscript.

## Discussion

All methods described above are in accordance with the *Cochrane Handbook for Systematic Reviews of Interventions* [25] and PRISMA-P guidance for reporting systematic review protocols [26,27], ensuring that our systematic review methodology is transparent, reproducible, and rigorous. The completed review will be reported in accordance with the PRISMA 2020 guidelines.

Recent systematic reviews and meta-analyses have addressed the effectiveness of neoadjuvant therapy in CCA and its impact on survival. A systematic review by Cremen et al [28] reported that neoadjuvant therapy followed by surgical resection was associated with improved patient outcomes and longer median overall survival compared to upfront surgery. Another systematic review comparing the same preoperative therapy followed by surgical resection to upfront surgery in patients with intrahepatic CCA found that neoadjuvant chemotherapy has the potential to improve 5-year overall survival without increasing complication rates [29]. However, the evidence remains limited as these systematic reviews are mostly based on retrospective studies and exhibit significant clinical heterogeneity. Cremen et al [28] could not proceed to meta-analysis due to significant heterogeneity in the available literature. However, we will be able to overcome this limitation by separately addressing the outcomes for patients with resectable and nonresectable CCA as several additional studies have been published in recent years.

Further subgroup analyses will be undertaken where data permit to explore statistical heterogeneity and clinically relevant differences between intrahepatic and extrahepatic CCA. Our preliminary search identified 2 phase 3 RCTs [30,31] and several recently published comparative studies evaluating neoadjuvant therapy in CCA. In addition, at least one further ongoing randomized trial has been identified through clinical trial registry searches [32] and is expected to be published within the anticipated review time frame. As these newer comparative trial data have not yet been fully integrated into the existing systematic review literature, their inclusion is expected to strengthen the evidence synthesis and improve the contemporary relevance of the review. Incorporating the most recent trial evidence may help generate more clinically meaningful conclusions regarding the role of neoadjuvant therapy in CCA and may better inform future practice and research.

## Supplementary material

10.2196/84912 Multimedia Appendix 1 MeSH (Medical Subject Headings) terms and keywords.

10.2196/84912 Checklist 1 PRISMA-P checklist.

## Abbreviations

CCA: cholangiocarcinoma
CR: complete response
GRADE: Grading of Recommendations Assessment, Development, and Evaluation
HR: hazard ratio
MD: mean difference
MeSH: Medical Subject Headings
PRESS: Peer Review of Electronic Search Strategies
PRISMA: Preferred Reporting Items for Systematic Reviews and Meta-Analyses
PRISMA-P: Preferred Reporting Items for Systematic Reviews and Meta-Analyses Protocols
RCT: randomized controlled trial
