# Supplementary material for: Safety and Efficacy of Neoadjuvant Therapy in Cholangiocarcinoma: Protocol for a Systematic Review and Meta-Analysis
Source: JMIR Res Protoc. 2026 Jun 10;15:e84912. doi: 10.2196/84912 (PMC13252700; doi:10.2196/84912)
Supplement: Multimedia Appendix 1 [file resprot-v15-e84912-s001.docx]

**Multimedia Appendix 1**

Database: Ovid MEDLINE(R) ALL <1946 to February 27, 2026>

Search Strategy:

1. Neoadjuvant Therapy/ (35155)
2. exp Neoadjuvant Therapy/ (35155)
3. exp Neoadjuvant Therapy/ (35155)
4. exp Neoadjuvant Therapy/ (35155)
5. Neoadjuvant.mp. [mp=title, book title, abstract, original title, name of substance word, subject heading word, floating sub-heading word, keyword heading word, organism supplementary concept word, protocol supplementary concept word, rare disease supplementary concept word, unique identifier, synonyms, population supplementary concept word, anatomy supplementary concept word] (68960)
6. cholangiocarcinoma.mp. [mp=title, book title, abstract, original title, name of substance word, subject heading word, floating sub-heading word, keyword heading word, organism supplementary concept word, protocol supplementary concept word, rare disease supplementary concept word, unique identifier, synonyms, population supplementary concept word, anatomy supplementary concept word] (24274)
7. exp Adenoma, Bile Duct/ or exp Cholangiocarcinoma/ or exp Bile Duct Neoplasms/ (27406)
8. Bile Duct Neoplasms/ (20468)
9. exp Bile Duct Neoplasms/ (24408)
10. exp Bile Duct Neoplasms/ or exp Cholangiocarcinoma/ or exp Biliary Tract Neoplasms/ (39330)
11. exp Biliary Tract Neoplasms/ (37385)
12. biliary tract cancer.mp. [mp=title, book title, abstract, original title, name of substance word, subject heading word, floating sub-heading word, keyword heading word, organism supplementary concept word, protocol supplementary concept word, rare disease supplementary concept word, unique identifier, synonyms, population supplementary concept word, anatomy supplementary concept word] (2964)
13. bile duct cancer.mp. [mp=title, book title, abstract, original title, name of substance word, subject heading word, floating sub-heading word, keyword heading word, organism supplementary concept word, protocol supplementary concept word, rare disease supplementary concept word, unique identifier, synonyms, population supplementary concept word, anatomy supplementary concept word] (1798)
14. bile duct tumour.mp. [mp=title, book title, abstract, original title, name of substance word, subject heading word, floating sub-heading word, keyword heading word, organism supplementary concept word, protocol supplementary concept word, rare disease supplementary concept word, unique identifier, synonyms, population supplementary concept word, anatomy supplementary concept word] (29)
15. 6 or 7 or 8 or 9 or 10 or 11 or 12 or 13 or 14 (49398)
16. Gemcitabine+capecitabine.mp. [mp=title, book title, abstract, original title, name of substance word, subject heading word, floating sub-heading word, keyword heading word, organism supplementary concept word, protocol supplementary concept word, rare disease supplementary concept word, unique identifier, synonyms, population supplementary concept word, anatomy supplementary concept word] (107)
17. GemCis.mp. [mp=title, book title, abstract, original title, name of substance word, subject heading word, floating sub-heading word, keyword heading word, organism supplementary concept word, protocol supplementary concept word, rare disease supplementary concept word, unique identifier, synonyms, population supplementary concept word, anatomy supplementary concept word] (60)
18. CisGem.mp. [mp=title, book title, abstract, original title, name of substance word, subject heading word, floating sub-heading word, keyword heading word, organism supplementary concept word, protocol supplementary concept word, rare disease supplementary concept word, unique identifier, synonyms, population supplementary concept word, anatomy supplementary concept word] (29)
19. Neoadjuvant Gemcitabine.mp. [mp=title, book title, abstract, original title, name of substance word, subject heading word, floating sub-heading word, keyword heading word, organism supplementary concept word, protocol supplementary concept word, rare disease supplementary concept word, unique identifier, synonyms, population supplementary concept word, anatomy supplementary concept word] (156)
20. Gem Cis durvalumab.mp. [mp=title, book title, abstract, original title, name of substance word, subject heading word, floating sub-heading word, keyword heading word, organism supplementary concept word, protocol supplementary concept word, rare disease supplementary concept word, unique identifier, synonyms, population supplementary concept word, anatomy supplementary concept word] (0)
21. GEMOX.mp. [mp=title, book title, abstract, original title, name of substance word, subject heading word, floating sub-heading word, keyword heading word, organism supplementary concept word, protocol supplementary concept word, rare disease supplementary concept word, unique identifier, synonyms, population supplementary concept word, anatomy supplementary concept word] (311)
22. Neoadjuvant Capecitabine.mp. [mp=title, book title, abstract, original title, name of substance word, subject heading word, floating sub-heading word, keyword heading word, organism supplementary concept word, protocol supplementary concept word, rare disease supplementary concept word, unique identifier, synonyms, population supplementary concept word, anatomy supplementary concept word] (51)
23. Neoadjuvant CapOx.mp. [mp=title, book title, abstract, original title, name of substance word, subject heading word, floating sub-heading word, keyword heading word, organism supplementary concept word, protocol supplementary concept word, rare disease supplementary concept word, unique identifier, synonyms, population supplementary concept word, anatomy supplementary concept word] (9)
24. 1 or 2 or 3 or 4 or 5 or 16 or 17 or 18 or 19 or 20 or 21 or 22 or 23 (69439)
25. 15 and 24 (956)

Database: Embase <1974 to 2026 Week 09>

Search Strategy:

1. exp neoadjuvant chemotherapy/ (50681)
2. exp neoadjuvant chemotherapy/ or exp neoadjuvant chemoradiotherapy/ or exp neoadjuvant therapy/ (81617)
3. exp bile duct carcinoma/ (49575)
4. cholangiocarcinoma.mp. [mp=title, abstract, heading word, drug trade name, original title, device manufacturer, drug manufacturer, device trade name, keyword heading word, floating subheading word, candidate term word] (37626)
5. exp bile duct cancer/ (55033)
6. biliary duct cancer.mp. [mp=title, abstract, heading word, drug trade name, original title, device manufacturer, drug manufacturer, device trade name, keyword heading word, floating subheading word, candidate term word] (63)
7. exp bile duct cancer/ or exp bile duct carcinoma/ or exp biliary tract cancer/ (72715)
8. exp biliary tract tumor/ or exp bile duct cancer/ or exp biliary tract cancer/ or exp bile duct tumor/ (81832)
9. biliary tract cancer.mp. [mp=title, abstract, heading word, drug trade name, original title, device manufacturer, drug manufacturer, device trade name, keyword heading word, floating subheading word, candidate term word] (9142)
10. neoadjuvant.mp. [mp=title, abstract, heading word, drug trade name, original title, device manufacturer, drug manufacturer, device trade name, keyword heading word, floating subheading word, candidate term word] (139951)
11. 3 or 4 or 5 or 6 or 7 or 8 or 9 (84896)
12. Gemcitabine+capecitabine.mp. [mp=title, abstract, heading word, drug trade name, original title, device manufacturer, drug manufacturer, device trade name, keyword heading word, floating subheading word, candidate term word] (268)
13. GemCis.mp. [mp=title, abstract, heading word, drug trade name, original title, device manufacturer, drug manufacturer, device trade name, keyword heading word, floating subheading word, candidate term word] (201)
14. CisGem.mp. [mp=title, abstract, heading word, drug trade name, original title, device manufacturer, drug manufacturer, device trade name, keyword heading word, floating subheading word, candidate term word] (81)
15. Neoadjuvant Gemcitabine.mp. [mp=title, abstract, heading word, drug trade name, original title, device manufacturer, drug manufacturer, device trade name, keyword heading word, floating subheading word, candidate term word] (319)
16. Gem Cis durvalumab.mp. [mp=title, abstract, heading word, drug trade name, original title, device manufacturer, drug manufacturer, device trade name, keyword heading word, floating subheading word, candidate term word] (1)
17. GEMOX.mp. [mp=title, abstract, heading word, drug trade name, original title, device manufacturer, drug manufacturer, device trade name, keyword heading word, floating subheading word, candidate term word] (1076)
18. Neoadjuvant Capecitabine.mp. [mp=title, abstract, heading word, drug trade name, original title, device manufacturer, drug manufacturer, device trade name, keyword heading word, floating subheading word, candidate term word] (106)
19. Neoadjuvant CapOx.mp. [mp=title, abstract, heading word, drug trade name, original title, device manufacturer, drug manufacturer, device trade name, keyword heading word, floating subheading word, candidate term word] (39)
20. 1 or 2 or 10 or 12 or 13 or 14 or 15 or 16 or 17 or 18 or 19 (141439)
21. 11 and 20 (2428)
