# Supplementary material for: Safety and Efficacy of Neoadjuvant Therapy in Cholangiocarcinoma: Protocol for a Systematic Review and Meta-Analysis
Source: JMIR Res Protoc. 2026 Jun 10;15:e84912. doi: 10.2196/84912 (PMC13252700; doi:10.2196/84912)
Supplement: Checklist 1 [file resprot-v15-e84912-s002.docx]

**PRISMA-P 2015 Checklist**

Safety and Efficacy of Neoadjuvant Therapy in Cholangiocarcinoma: a Systematic Review and Meta-analysis Protocol

| **Section/topic** | **Item** | **Category** | **Checklist item** | **Location in Protocol** |
| --- | --- | --- | --- | --- |
| Administrative information | 1a | Identification | Identify the report as a protocol of a systematic review. | Title |
| Administrative information | 1b | Update | If the protocol is for an update of a previous systematic review, identify as such. | Not applicable |
| Administrative information | 2 | Registration | If registered, provide the name of the registry and registration number. | Abstract; Methods |
| Administrative information | 3a | Contact | Provide name, institutional affiliation, and email address of all protocol authors; provide physical mailing address of corresponding author. | Title page |
| Administrative information | 3b | Contributions | Describe contributions of protocol authors and identify the guarantor of the review. | Author Contribution declaration |
| Administrative information | 4 | Amendments | If the protocol amends a previously completed or published protocol, identify as such and list changes; otherwise state how important amendments will be documented. | Methods (opening paragraph after heading) |
| Administrative information | 5a | Sources | Indicate sources of financial or other support for the review. | Funding |
| Administrative information | 5b | Sponsor | Provide the name of the review funder and/or sponsor. | Funding |
| Administrative information | 5c | Role of sponsor or funder | Describe roles of funder(s), sponsor(s), and/or institution(s), if any, in developing the protocol. | Funding |
| Introduction | 6 | Rationale | Describe the rationale for the review in the context of what is already known. | Introduction |
| Introduction | 7 | Objectives | Provide an explicit statement of the question(s) the review will address with reference to participants, interventions, comparators, and outcomes (PICO). | Objectives; Methods |
| Methods | 8 | Eligibility criteria | Specify study characteristics and report characteristics to be used as criteria for eligibility for the review. | Methods > Types of studies; Types of participants; Types of interventions |
| Methods | 9 | Information sources | Describe all intended information sources, with planned dates of coverage. | Methods > Electronic searches; Searching other resources; Appendix 1 |
| Methods | 10 | Search strategy | Present draft of search strategy to be used for at least one electronic database, including planned limits, such that it could be repeated. | Appendix 1 |
| Methods | 11a | Data management | Describe the mechanism(s) that will be used to manage records and data throughout the review. | Data extraction and management |
| Methods | 11b | Selection process | State the process that will be used for selecting studies through each phase of the review. | Selection of studies |
| Methods | 11c | Data collection process | Describe planned method of extracting data from reports. | Data extraction and management |
| Methods | 12 | Data items | List and define all variables for which data will be sought. | Data extraction and management |
| Methods | 13 | Outcomes and prioritization | List and define all outcomes for which data will be sought, including prioritization of main and additional outcomes, with rationale. | Types of outcome measure |
| Methods | 14 | Risk of bias in individual studies | Describe anticipated methods for assessing risk of bias of individual studies. | Assessment of risk of bias in included studies |
| Methods | 15a | Data synthesis | Describe criteria under which study data will be quantitatively synthesized. | Assessment of heterogeneity; Data synthesis |
| Methods | 15b | Data synthesis | If data are appropriate for quantitative synthesis, describe planned summary measures, methods of handling data, and methods of combining data from studies, including planned exploration of consistency. | Measures of treatment effect; Data synthesis |
| Methods | 15c | Data synthesis | Describe any proposed additional analyses (such as sensitivity or subgroup analyses, meta-regression). | Subgroup analysis and investigation of heterogeneity; Sensitivity analysis |
| Methods | 15d | Data synthesis | If quantitative synthesis is not appropriate, describe the type of summary planned. | Data synthesis |
| Methods | 16 | Meta-bias(es) | Specify any planned assessment of meta-bias(es), such as publication bias across studies or selective reporting within studies. | Assessment of reporting biases |
| Methods | 17 | Confidence in cumulative evidence | Describe how the strength of the body of evidence will be assessed. | Data synthesis |

Item 1b is not applicable because this manuscript is not an update of a previously completed systematic review.
